# Effects of Qigong on Depression: A Systemic Review

**DOI:** 10.1155/2013/134737

**Published:** 2013-03-04

**Authors:** Byeongsang Oh, Sun Mi Choi, Aya Inamori, David Rosenthal, Albert Yeung

**Affiliations:** ^1^Dana-Farber Cancer Institute and Harvard Medical School, 450 Brookline Avenue, Boston, MA 02215, USA; ^2^Sydney Medical School, University of Sydney, Edward Ford Building A27, Fisher Road, Camperdown, NSW 2006, Australia; ^3^Korea Institute of Oriental Medicine, Acupuncture, Moxibustion & Meridian Research Centre, 1672 Yuseongdae-ro, Yuseong-gu, Daejeon 305-811, Republic of Korea; ^4^Massachusetts General Hospital and Harvard Medical School, 55 Fruit Street, Boston, MA 02114, USA

## Abstract

Physical exercises and relaxation have been found to be beneficial for depression. However, there is little evidence on the use of Qigong, a mind-body practice integrating gentle exercise and relaxation, in the management of depression. The aim of this paper is to evaluate the effects of Qigong on depression. The paper examined clinical trials measuring the effect of Qigong on depression within six large-scale medical research databases (PubMed, Medline, ProQuest, Science Direct, EMBASE, and PsycInfo) till October 2011. Key words “Qigong,” “depression,” and “mood” were used. Ten studies were identified as original randomized controlled trial (RCT) studies investigating the effect of Qigong on depression as primary (*n* = 2) or secondary outcome (*n* = 8). Four studies reported positive results of the Qigong treatment on depression; two reported that Qigong effect on depression was as effective as physical exercise. One study reported that Qigong was comparable to a conventional rehabilitation program, but the remaining three studies found no benefits of Qigong on depression. While the evidence suggests the potential effects of Qigong in the treatment of depression, the review of the literature shows inconclusive results. Further research using rigorous study designs is necessary to investigate the effectiveness of Qigong in depression.

## 1. Introduction

Depression is a common illness occurring in approximately 5–13% of women and 2–8% of men at any particular point in time. Depression has a lifetime prevalence rate of 16.2% and a twelve-month prevalence rate of 6.6% [1, 2]. By 2020, depression is projected to become the second global leading cause of disability [3]. The annual cost of depression in the United States exceeds $80 billion, surpassing that of other chronic illnesses such as diabetes and hypertension [4]. Depression not only accounts for up to 70% of psychiatric hospitalizations and 60% of suicides, but also complicates the management of other diseases [5]. Despite the growing number of marketed antidepressants, between 19 and 34% of patients with depression do not respond to acute antidepressant treatment. 29–46% of patients with depression fail to achieve full remission, and up to 50% of patients experience recurrence [6, 7]. Given the scale of this problem, there is a need to explore alternative and complementary forms of treatment. In recent years, there has been a growing interest in alternative medical approaches to treating depression, including acupuncture [8, 9], Tai Chi [10], meditation [11, 12], and Qigong [13, 14]. 

Qigong is a traditional Chinese mind-body medicine dating back to over one thousand years. It consists of two types: internal and external Qigong. Internal Qigong is a form of mind-body medicine that involves coordinated gentle exercise and relaxation through meditation and breathing [15]. The practice of internal Qigong promotes balance and is believed to combat energy blockages by facilitating the flow of vital energy around the body [16]. In doing so, it contributes to both physical and psychological well-being. External Qigong, on the other hand, is a branch of energy medicine in which an experienced Qigong practitioner sends or emits Qi—a form of energy—to a patient for the treatment of that patient's illnesses [17]. 

Existing literature has reported that internal Qigong decreases heart rate [18], blood pressure [19], lipid levels [19], and circulating stress hormones [20] as well as improves the body's immune function [20, 21]. Moreover, a recent review which examined 77 articles on the physiological and psychological effects of Tai Chi and Qigong suggests that both Tai Chi and Qigong have beneficial effects on bone density, cardiopulmonary functions, physical and immune functions, self-efficacy, and quality of life and improve psychological symptoms [22]. Most of the earlier studies on Qigong recruited subjects with a variety of medical conditions; however, only a few specifically examined the effects of Qigong on patients with depression. This paper aims to fill the gap in the literature by examining the reported effects of internal Qigong on depression and demonstrates the need for further research. 

## 2. Methods

A literature search reviewing all published articles prior to October 2011 on the effect of Qigong on depression was conducted using PubMed, Medline (1950~), ProQuest (1950~), ScienceDirect (1950~), EMBASE, and PsycINFO (1806~). Key words “Qigong,” “depression,” and “mood” were used in the literature search. Identified records were initially screened for eligibility based on title and abstract. Reference lists of identified papers and reviews were manually searched for additional studies in related areas. Articles were finally selected based on the analysis of the full text. Two reviewers, B. Oh, and A. Yeung, independently applied the inclusion criteria. The two reviewers compared results and resolved any discrepancies by agreement.

## 3. Eligibility Criteria

Studies which investigated the effects of Qigong on depression as primary or secondary outcomes based on randomized controlled trial (RCT) design were eligible. Articles reporting on interventions using external Qigong or Tai Chi were excluded. The literature search included only papers with the full article published in English. 

## 4. Results

**Figure 1:**
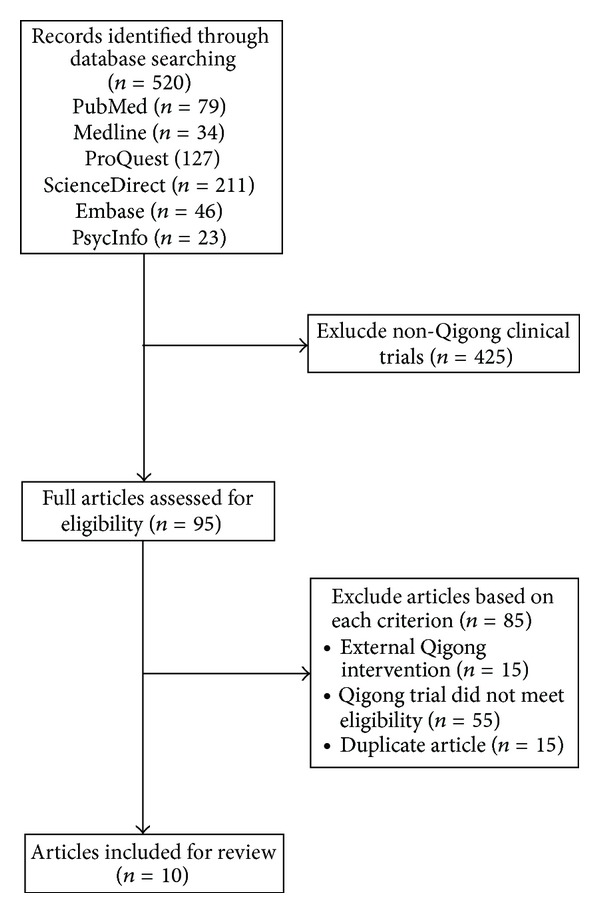
Flowchart of review process.

The initial literature search identified 520 articles using the key words “Qigong,” “depression,” and “mood,” of which 425 non-Qigong clinical trial articles were excluded. Of the 95 remaining articles, 10 articles met the eligibility criteria and were reviewed, as shown in Figure 1. Of the ten studies shown in Table 1, two studies measured depression as a primary outcome [13, 23] and eight studies measured depression as a secondary outcome. The former two studies measured the effects of Qigong on geriatric depression. 

In one of these two studies, Tsang et al. [23] showed that Qigong was as effective as a conventional rehabilitation program in a pilot study. In a subsequent study with a larger sample size, Tsang et al. [13] demonstrated a significant difference between the Qigong intervention and the control group. The remaining eight studies measured depression as a secondary outcome among patients who were recruited based on other medical conditions. Of eight studies, three studies reported a significant difference between Qigong intervention and control groups [24–26]. Two studies showed that Qigong was as effective as physical exercise [27, 28]. Three studies did not find a significant difference between the Qigong intervention and the control group [29–31]. 

These studies were conducted in Hong Kong (*n* = 3), Korea (*n* = 1), Germany (*n* = 2), Sweden (*n* = 2), Australia (*n* = 1), and the USA (*n* = 1) between 2003 and 2009. Among them, there were ten RCTs, which employed two arms (Qigong versus control group). Six of these ten studies used a sham intervention for the control groups. None of the studies used a double-blind design. 

Sample sizes ranged from 50 to 162 subjects. Study populations varied, with two studies conducted on geriatric patients with depression. Other study populations included patients with fibromyalgia, hypertension, Parkinson's disease, cancer, burnout, severe chronic pain, and female college students. One study did not report a detailed demographic profile of its participants, as the study was conducted during a Qigong retreat [32]. The mean age of participants ranged from 19 to 82. 

The duration of Qigong interventions also varied across studies. Qigong intervention period ranged from 6 to 16 weeks (6 weeks (*n* = 1), 8 weeks (*n* = 2), 12 weeks (*n* = 4), and 16 weeks (*n* = 2)), and one study involved a four-day Qigong retreat. The duration of each intervention also varied from 30 minutes (*n* = 1), 45 minutes (*n* = 2), 60 minutes (*n* = 3), and 90 minutes (*n* = 2) to 120 minutes (*n* = 1). Most studies (*n* = 7) offered Qigong interventions twice a week. One study [32] did not describe the length or frequency of the intervention. Three out of the 10 studies had long-term follow-up assessments at 24 weeks [29], 6 months [28], and 12 months [30].

The ten studies used different instruments to measure depression outcomes. Two studies used the Geriatric Depression Scale (GDS), two used the Beck Depression Inventory (BDI), and two used the Profile of Mood States (POMS). Other instruments included the Depression Status Inventory (DSI), Montgomery-Asberg Depression Rating Scale (MADRS), Hospital Anxiety and Depression Scale (HADS), and Allgemeine Depressionsskala (ADS). 

With the exception of one study that had an unusually high dropout rate of 49% [29], most studies had satisfactory dropout rates ranging from 3% to 24%. Two studies did not report their dropout rates [23, 24]. Two studies [27, 28] reported adverse events that were not directly related to the practice of Qigong; eight studies did not report adverse events; and one reported no adverse effects of Qigong intervention [26]. Two studies which recruited patients with hypertension and chronic neck pain reported adverse events not specifically related to the intervention, such as muscle ache, tension, nausea, and vestibular neuronitis. 

Of the ten studies, five conducted intention-to-treat (ITT) statistical analyses, two conducted completers analyses [25, 29], and one performed both ITT and completer analyses [31]. Two studies did not report the details of their data analysis methods [13, 23]. 

## 5. Discussion 

This paper suggests that the effect of Qigong in the treatment of depression is inconclusive, although potential effects of Qigong in the treatment of depression was supported by the biopsychosocial model [33], relaxation response theory [34, 35], and evidence on the positive effects of exercise [36, 37]. Further, our review result was not consistent with the previous review conducted by Jahnke et al., which reported the effect of Qigong and Tai Chi in the treatment of psychological symptoms including depression [22]. Their review concluded that Qigong and Tai Chi can reduce psychological symptoms including depression. The differences in results may be due to the different inclusion criteria used in the two reviews. In Jahnke et al.'s review [22], their results were based more on tai chi studies than on Qigong studies, while our review assessed exclusively Qigong studies. Although, tai chi is considered as part of moving Qigong, the differences and similarities of basic philosophy and practices between tai chi and Qigong are debated among the academic researchers, particularly in Western world [22, 38]. Interestingly, the result of this review is similar to a recent review conducted by Tsang et al. with 12 RCTs which compared the effects of mindful exercises versus nonmindful exercises [39]. This review reported that both mindful and nonmindful physical exercises were effective in the treatment of depression or depressive symptoms in the short term. Our paper also showed that Qigong was as effective as physical exercise and rehabilitation program for treatment of depression. Results from Jahnke et al. [22] and our review are compatible with two recent reviews on the efficacy of exercise on depression, both concluding that exercise has a mild treatment effect on depression [36, 37]. These findings suggest that future studies are needed to examine the mechanism of the effects of Qigong, tai chi, and physical exercise on the brain to decipher the similarities and differences of their effects on depression. 

One of most remarkable findings of this review (*n* = 10) was that participants did not report any psychotic reactions from Qigong, as previous literature has indicated as possible concern [25]. The inconsistent results based on the above reviews may reflect dose response of subjects receiving Qigong intervention with different frequencies, lengths, and intensities. A limitation of this review is that it included studies with small sample sizes and no appropriate sham intervention for control group, no blinding of subjects and Qigong instructors, and they used various instruments to measure depression outcomes. There was moreover a publication selection bias, as we only examined studies published in English. 

Future studies may take into account the following suggestions for methodology. First, participants who meet the criteria for major depressive disorder, dysthymic disorder, or depressive disorder not otherwise specified based on DSM-IV criteria should be included. Since Qigong originated in the East, different ethnic groups may be recruited to examine if cultural differences could be a mediator or moderator of treatment outcomes. A three-arm design (Qigong intervention versus sham Qigong versus usual care or waitlist group) with adequate sample size is recommended to detect statistical and clinical significance, as suggested by Oh et al. [26]. Dose-response relationship can be examined by varying length (e.g., 30 minutes versus 60 minutes versus 90 minutes), frequency (e.g., weekly versus biweekly versus every 4 weeks), and intensity of intervention, as measured by physical activity intensity scale. Both quantitative and qualitative outcome measures are recommended in order to capture the complexity of depression treatment effect. Measureing of biomarkers, such as immune function, cytokines, and DNA damage level, may provide objective information on the physiological and psychological effects of Qigong intervention. Finally, a cost-benefit analysis could examine possible health policy considerations. 

In conclusion, all studies suggest that Qigong intervention for patients with major depressive disorder is safe and feasible; however, evidence for its effectiveness is limited. Future study with more robust design is warranted. 

## Figures and Tables

**Table 1:** Two studies investigating the effects of Qigong on depression as primary and eight studies as secondary outcomes.

Author Year Country	Design Blinding	Sample size	Subject Mean age Sex (m/f)	Intervention control	Outcome measure Followup Analysis	Result Dropout	Adverse event	Conclusion and discussion
Tsang et al. [23] 2003 Hong Kong	RCT Non-blinding	Total (*n* = 50) Intervention (*n* = 25) Control (*n* = 25)	Geriatric patients 74 (26/24)	Eight-section brocades Qigong (a) 12 weeks (b) 60 minutes (c) 6 visits (d) 2x/week Traditional remedial rehabilitation activities	(1) Geriatric Depression Scale (2) Perceived Benefit Questionnaire (3) Hong Kong Chinese Version World Health Organization Quality of Life (4) Self-concept Scale No follow-up	(1) NS (2) *P* < 0.001 (3) NS (4) NS n/a	Not reported	Eight section brocades Qigong is promising as an alternative psychosocial intervention for depressed elderly with chronic physical illness. Although there is no evidence, there is an optimistic stance that Qigong results in better treatment compliance and better outcome compared to Western exercise protocols like aerobics

Astin et al. [29] 2003 USA	RCT Non-blinding	Total (*n* = 128) Intervention (*n* = 64) Control (*n* = 64)	Patients with fibromyalgia syndrome 48 (1/127)	Mindfulness meditation with Qigong movement therapy (a) 8 weeks (b) 150 minutes (90 min mindfulness, 60 min Qigong) (c) 8 visits (d) 1x/week Education support group	(1) Pain measured with 36-Item Short-Form Health Survey (2) Fibromyalgia Impact Questionnaire (3) Beck Depression Inventory (4) Myalgic score (5) Coping strategies Follow-up (a) Week 4 (b) Week 24 Completers analysis	(1) NS (2) NS (3) NS (4) NS (5) NS 49%	Not reported	Both intervention and control groups showed improvement on a number of outcome variables, however, no evidence showed that the mindfulness meditation and Qigong intervention for fibromyalgia was superior to education support group.

Kim et al. [24] 2004 Korea	RCT Non-blinding	Total (*n* = 54) Intervention (*n* = 26) Control (*n* = 28)	Female college students 19–21 (0/54)	Meridian exercise (a) 6 weeks (b) 30 minutes (c) 12 visits (d) 2x/week Standard care only	(1) State Anxiety Inventory (2) Depression Status Inventory (3) Self-Esteem Inventory No followup: ITT analyses	(1) *P* < 0.001 (2) *P* < 0.001 (3) *P* < 0.001 n/a	Not reported	Meridian exercise decreased anxiety and depression and increased self-esteem. Study suggests that meridian exercise enabled female students to manage their mental health within the community. Future studies are needed to examine the lasting effect of the intervention, including physiological indices

Cheung et al. [27] 2005 Hong Kong	RCT Non-blinding	Total (*n* = 91) Intervention (*n* = 47) Control (*n* = 44)	Patients with essential hypertension 54 (37/51)	Guolin Qigong (a) 4 weeks (b) 120 minutes (c) 8 visits (d) 2x/week Conventional exercise	(1) Blood pressure (2) 36-Item Short-Form Health Survey (3) Beck Anxiety Inventory (4) Beck Depression Inventory No followup ITT analyses	(1) NS (2) NS (3) NS (4) NS 16.5%	Vestibular neuronitis, unrelated to Qigong practice	Goulin Qigong and conventional exercise had similar effects on blood pressure in patients with mild hypertension. While no additional benefits were identified, Qigong treatment serves as a nondrug alternative to conventional exercise in the treatment of hypertension

Tsang et al. [13] 2006 Hong Kong	RCT Single blind	Total (*n* = 82) Intervention (*n* = 34) Control (*n* = 48)	Geriatric patients 82 (16/66)	Baduanjin Qigong (a) 16 weeks (b) 30–45 minutes (c) 48 visits (d) 3x/week Newspaper reading	(1) Geriatric Depression Scale (2) Chinese General Self-Efficacy Scale (3) Personal Well-Being Index (4) General Health Questionnaire-12 (5) Self-Concept Scale (6) Perceived Benefit Questionnaire Followup: (a) Week 4 (b) Week 8	(1) *P* = 0.041 (2) *P* < 0.001 (3) *P* < 0.001 (4) *P* = 0.042 (5) Subscales significant (6) *P* < 0.001 15.8%	Not reported	Regular Qigong practice could reduce depression, and improve self-efficacy and personal well-being among geriatric patients with chronic physical illness and depression. Study shows that practice needs to continue and last for long-term effects

Schmitz-H$\ddot{\text{u}}$bsch et al. [30] 2006 Germany	RCT Non-blinding Pilot study	Total (*n* = 56) Intervention (*n* = 32) Control (*n* = 24)	Patients with Parkinson's Disease 63 (43/13)	Qigong (frolic of the crane, eight-section brocades in sitting position) (a) 24 weeks (8-week intervention, 8-week no intervention, 8-week intervention) (b) 90 minutes (c) 16 visits (d) 1x/week No intervention	(1) Unified Parkinson's Disease Rating Scale-Motor (2) Parkinson's Disease Questionnaire (3) Montgomery-Asperg Depression Rating Scale Followup: (a) 12 months ITT analyses	(1) *P* = 0.008 (2) NS (3) NS 12.5%	Not reported	Results suggest positive effects of Qigong on symptoms of autonomic dysfunction in patients with Parkinson's disease. Given high acceptance and compliance with therapy, Qigong is a promising treatment with possible effects on motor as well as nonmotor symptoms. Group instruction, as well as self-exercise of Qigong, moreover serves as cost-effective application

Johansson et al. [25] 2008 Sweden	RCT Non-blinding	Total (*n* = 59) Intervention (*n* = 28) Control (*n* = 31)	Summer school camp participants 51 (8/51)	Jichu Gong (a) 4-day retreat Lecture on Chinese medicine	(1) Profile of Mood Status (2) State and Trait Anxiety Inventory No followup Completers analyses	(1) *P* < 0.0005 (2) *P* < 0.0005 3%	Not reported	Study supports the effectiveness of Qigong to promote mental health. More studies are necessary to verify the finding

Oh et al. [26] 2009 Australia	RCT Non-blinding	Total (*n* = 162) Intervention (*n* = 79) Control (*n* = 83)	Cancer patients 60 (69/93)	Medical Qigong + standard care (a) 12 weeks (b) 90 minutes (c) 12 visits (d) 1x/week Standard care only	(1) Functional Assessment of Cancer Therapy-General (2) Functional Assessment of Cancer Therapy-Fatigue (3) Profile of Mood Status (4) Inflammation (CRP) No followup ITT analyses	(1) *P* < 0.01 (2) *P* < 0.01 (3) *P* = 0.021 (mood disturbance) *P* = 0.0290 (depression) (4) *P* = 0.044 24%	Reported no adverse event	Medical Qigong can improve cancer patients overall quality of life and mood status as well as reduce specific side effects of cancer treatment. Qigong treatment may also produce long-term physical benefits due to reduction of CRP inflammation

Stenlund et. al. [31] 2009 Sweden	RCT Non-blinding	Total (*n* = 82) Intervention (*n* = 41) Control (*n* = 41)	Patients with burnout	Qigong + basic care (a) 12 weeks (b) 60 minutes (c) 12 visits (d) 1x/week Basic care at the stress clinic	(1) Shirom-Melamed Burnout Questionnaire (2) 36-Item Short Form Health Survey (Swedish) (3) Self-Concept Questionnaire (4) Checklist Individual Strength Questionnaire (5) Hospital Anxiety and Depression Scale (6) Physical Assessment Scale of the Relaxation Inventory No followup ITT analyses Completers analyses	(1) NS (2) NS (3) NS (4) NS (5) NS (6) NS 17%	Not reported	12-week intervention of Qigong had no additional benefit compared to basic care in burnout patients
